# Cellular Internalization Mechanism and Intracellular Trafficking of Filamentous M13 Phages Displaying a Cell-Penetrating Transbody and TAT Peptide

**DOI:** 10.1371/journal.pone.0051813

**Published:** 2012-12-14

**Authors:** Aeyung Kim, Tae-Hwan Shin, Seung-Min Shin, Chuong D. Pham, Dong-Ki Choi, Myung-Hee Kwon, Yong-Sung Kim

**Affiliations:** 1 Department of Molecular Science and Technology, Ajou University, Suwon, Korea; 2 Department of Microbiology, Ajou University School of Medicine, Suwon, Korea; Consejo Superior de Investigaciones Cientificas, Spain

## Abstract

Cellular internalization of bacteriophage by surface-displayed cell penetrating peptides has been reported, though the underlying mechanism remains elusive. Here we describe in detail the internalization mechanism and intracellular trafficking and stability of filamentous M13 phages, the cellular entry of which is mediated by surface-displayed cell-penetrating light chain variable domain 3D8 VL transbody (3D8 VL-M13) or TAT peptide (TAT-M13). Recombinant 3D8 VL-M13 and TAT-M13 phages were efficiently internalized into living mammalian cells via physiologically relevant, energy-dependent endocytosis and were recovered from the cells in their infective form with the yield of 3D8 VL-M13 being higher (0.005∼0.01%) than that of TAT-M13 (0.001∼0.005%). Biochemical and genetic studies revealed that 3D8 VL-M13 was internalized principally by caveolae-mediated endocytosis via interaction with heparan sulfate proteoglycans as cell surface receptors, whereas TAT-M13 was internalized by clathrin- and caveolae-mediated endocytosis utilizing chondroitin sulfate proteoglycans as cell surface receptors, suggesting that phage internalization occurs by physiological endocytotic mechanism through specific cell surface receptors rather than non-specific transcytotic pathways. Internalized 3D8 VL-M13 phages routed to the cytosol and remained stable for more than 18 h without further trafficking to other subcellular compartments, whereas TAT-M13 phages routed to several subcellular compartments before being degraded in lysosomes even after 2 h of internalization. Our results suggest that the internalizing mechanism and intracellular trafficking of filamentous M13 bacteriophages largely follow the attributes of the displayed cell-penetrating moiety. Efficient internalization and cytosolic localization of 3D8 VL transbody-displayed phages will provide a useful tool for intracellular delivery of polar macromolecules such as proteins, peptides, and siRNAs.

## Introduction

Eukaryotic endocytosis of exogenous proteins, toxins, bacteria, and viral particles occurs through a vesicular transport mechanism facilitating transfer of the polar macromolecules across non-polar, lipophilic cell membranes. The best-studied mechanisms are clathrin-mediated endocytosis (CME), caveolae-mediated (caveolar/lipid raft-dependent) endocytosis, and macropinocytosis [1], [2]. There are, in addition, several clathrin- and caveolin-independent mechanisms that are less well characterized [2]. The main differences between these endocytotic mechanisms are the size of the vesicles formed upon internalization, associated molecular machinery, and the intracellular trafficking route, the details of which are not yet clear [1].

Cell-penetrating peptides (CPPs), such as the TAT peptide derived from HIV-1 transactivator protein, have the ability to penetrate into living mammalian cells [3], [4]. Because of their cationic nature, cationic CPPs, including TAT, utilize negatively charged glycosaminoglycans (GAGs) such as heparan sulfate (HS) and chondroitin sulfate (CS) as cell surface receptors for the cellular uptake [5]. GAGs are covalently linked to cell surface core proteins such as syndecan and glypican forming HS proteoglycan (HSPG) and CS proteoglycan (CSPG) [6]. These anionic proteoglycans due to abundant carboxyl and sulfate groups in HS and CS are present on the surface of virtually all mammalian cells, explaining the cell type-independent internalizing ability of cationic CPPs via electrostatic interactions [3]. Despite consensus on sulfated proteoglycans as the cell surface receptor, the endocytosis of the TAT peptide remains controversial [4] and has been proposed by several mechanisms, such as clathrin-mediated endocytosis [7], caveolae-mediated endocytosis [8], or macropinocytosis [5].

Some cationic proteins, such as anti-DNA antibodies with an unusually high number of basic amino acids in the complementarity-determining regions (CDRs) [9] and naturally occurring human proteins with a high net positive charge [10], also internalize into living cells. These polybasic proteins also utilize anionic sulfated proteoglycans as cell surface receptors for the cellular internalization [11]. We previously documented a cell-penetrating antibody (transbody) anti-DNA 3D8, which binds to DNA and RNA without sequence specificity [12]. In the format of single chain variable fragment (scFv) and light chain variable single domain (VL), 3D8 transbody efficiently internalizes into living cells by caveolae-mediated endocytosis through electrostatic interactions with HSPG [13], [14], [15], [16].

Phage display is the most widely used technology for the display of antibody, protein, and peptide libraries, from which target-specific binders can be isolated and engineered. Furthermore, the phage displaying integrin-binding cyclic RGD peptide not only bound to integrin-expressing cells and underwent internalization into the cells [17], but also could be recovered in an infective form by cell lysis for amplification [18]. Thereafter, phage peptide and antibody libraries have been extensively exploited to isolate novel binders to endocytosed receptors for internalization into target cells [19], [20], [21], [22]. More interestingly, TAT peptide displayed on the surface of bacteriophage facilitated cellular internalization of the recombinant phage into mammalian cells [20], [23], [24]. For example, phage-surface displayed TAT peptide enabled the large bacteriophages such as filamentous M13 (approximately 900 nm in length and 9 nm in width) and tailored λ phages (head, 60 nm; tail, 150 nm in diameter) to internalize into various tumor cells [23], [24]. CPP-mediated phage internalization has also been exploited to promote efficient gene delivery into mammalian cells [25], [26].

Despite the broad applications of phage internalization by displaying various cellular-internalizing moieties of peptides and antibody, however, the endocytosis process has not been characterized in detail. There have been no studies on whether the internalizing phages by surface-displayed CPPs also use HSPG and CSPG as cell surface receptors. As phage particles are large in comparison to free peptides and proteins, the cell entry mechanism of phages has been proposed to involve a phagocytosis-like process [17] or an energy-independent transcytotic pathway that occurs even at 4°C [23]. Furthermore, there have been no comparative studies on phage internalization depending on the different cell-penetrating moiety displayed on the phage surface.

We investigated the internalization mechanism and intracellular trafficking and stability of filamentous M13 phages, which were enabled to internalize into living mammalian cells by displaying the cell-penetrating 3D8 VL transbody (3D8 VL-M13) or TAT peptide (TAT-M13) on the surface via fusion with the minor coat protein pIII (g3p). The internalized recombinant phages could be rescued in the infective form by cell lysis, allowing quantification by infection into bacteria. We found that the cellular internalization of recombinant 3D8 VL-M13 and TAT-M13 phages involves physiological endocytosis rather than non-specific transcytotic pathways and is mediated by sulfated proteoglycans as cell surface receptors. However, the internalization mechanism, cell surface receptors and intracellular trafficking of 3D8 VL-M13 and TAT-M13 are different each other, resulting in distinct intracellular stability. Our comparative study of 3D8 VL-M13 and TAT-M13 provides a deep understanding of the cellular internalization of filamentous phages into mammalian cells and their intracellular fates.

## Materials and Methods

### Materials

*Escherichia coli* strains ER2738 and XL1-Blue, bacterial hosts for M13 phage, were obtained from New England BioLabs (Beverly, MA). Chlorpromazine (CPZ), methyl-β-cyclodextrin (MβCD), cytochalasin D (Cyt-D), isopropyl-b-D-1-thiogalactoside (IPTG), soluble GAGs (heparin from porcine intestinal mucosa, chondroitin sulfate A from bovine trachea, chondroitin sulfate B from porcine intestinal mucosa, and chondroitin sulfate C from shark cartilage), GAG lysases (heparinase III from *Flavobacterium heparinum* and chondroitinase ABC from *Proteus vulgaris*), and fluorescein isothiocyanate (FITC)-goat anti-mouse antibody were purchased from Sigma (St Louis, MO). Alexa 488-transferrin (TF), Alexa 488-cholera toxin B (Ctx-B), FITC-dextran, and LysoTracker®Red DND-99 were obtained from Invitrogen-Molecular Probes (Eugene, OR). Tetramethyl-rhodamine isothiocyanate (TRITC)-goat anti-mouse antibody, horse radish peroxidase (HRP)-conjugated anti-mouse antibody, anti-myc, anti-M13 major coat protein (anti-pVIII), anti-caveolin-1, anti-clathrin, anti-EEA-1, anti-calnexin, and anti-58K Golgi protein antibodies were obtained from Santa Cruz Biotechnology lnc. (Santa Cruz, CA). Anti-M13 pIII (g3p) antibody was purchased from New England BioLabs and anti-dynamin antibody was from Stressgene (Ann Arbor, MI).

### Cell Lines

Human cervical carcinoma HeLa, breast carcinoma MDA-MB-231, and melanoma A375-SM cells were purchased from American Type Culture Collection (ATCC, Manassas, VA) and were maintained in Dulbecco’s Modified Eagle Medium (DMEM; Welgene, Korea) or minimum essential medium (MEM; Welgene). Wild-type Chinese hamster ovary (CHO)-K1 and the proteoglycan-deficient CHO-K1 mutant cell lines, pgsA-745 and pgsD-677, were purchased from ATCC, and maintained in F-12K Nutrient Mixture (Kaighn’s modification of Ham’s F-12 Medium; GIBCO/Invitrogen). The pgsA-745 cell line lacks xylosyltransferase, an enzyme that initiates glycosaminoglycan (GAG) synthesis, causing a defect in HS and CS [27], [28]. The pgsD-677 has a single mutation that impairs both *N*-acetylglucosaminyltransferase and glucuronosyltransferase activities, which are essential for the polymerization of HS disaccharide chain, causing selective depletion of HS without affecting CS expression [27]. All cells were cultured in media supplemented with 10% (v/v) heat-inactivated fetal bovine serum (FBS; GIBCO/Invitrogen, Carlsbad, CA), 100 units/ml penicillin, and 100 µg/ml streptomycin (Welgene) at 37°C in a humidified 5% CO_2_ incubator.

### Construction of Recombinant Phagemids by Fusion with pIII Protein

The pIII-display phagemid pDR-D1 vector, a gift from Dr. Sang-Jick Kim (KRIBB, Korea), has the carboxy-terminal domain (residues 230–406) of pIII in downstream of the PelB secretion leader sequence with a myc tag sequence (EQKLISEEDL) between the insert and pIII sequences and a 6×His tag in the C-terminus of pIII (PelB-*Sfi*I-insert-*Sfi*I-myc-*Not*I-pIII-*Not*I-6×his) under the control of lac promoter [29] (Figure S1A). The open reading frame of 3D8 VL transbody [13], [14], TAT peptide (residues 48–60, GRKKRRQRRRPPQ) [23], and anti-death receptor 4 (DR4, TRAIL receptor 1) hAY4 scFv [30] was subcloned with *Sfi*I into the N-terminus of pIII, resulting in fusion phagemids encoding 3D8 VL-pIII, TAT-pIII, and hAY4 scFv-pIII fusion proteins, respectively (Figure S1A).

### Phage Growth, Purification, and Titration

VCSM13 helper phage (Stratagene, La Jolla, CA) was prepared as previously described [29]. The recombinant phagemid vectors were electroporated into *E. coli* ER2738 cells. Recombinant phages displaying the insert-pIII were recovered by superinfection of phagemid-bearing bacteria with the VCSM13 helper phage at a multiplicity of infection (MOI) of 20∶1 (phage-to-cell ratio) [29], [31]. Briefly, ER2738 cells carrying fusion phagemids were grown at 37°C to mid-log phase (OD_600_ of 0.6–0.8) in 2×YT medium containing 100 µg/ml ampicillin and 2% glucose (2×YT/AG) medium. Bacteria were pelleted by centrifugation at 2500×g for 5 min, and resuspended with 10 ml fresh 2×YT medium containing 2% glucose (2×YT/G). After addition of VCSM13 helper phage onto the cell suspension at a MOI of 20, cells were incubated for 30 min at 37°C without shaking and for another 30 min with shaking at 180 rpm. The cells and helper phage mixtures were centrifuged at 2500×g for 5 min, and the supernatants were discarded. The cell pellets were resuspended in 100 ml fresh 2×YT medium containing 100 µg/ml ampicillin, 50 µg/ml kanamycin, and 5 µg/ml tetracyclin (2×YT/AKT), and then phages were propagated by growing the cells at 20°C and 120 rpm without IPTG for 20 h for 3D8 VL and hAY4 scFv display or by addition of 0.5 mM IPTG and growth at 37°C and 180 rpm for 20 h for TAT display, unless otherwise specified. Recombinant phage particles were harvested by centrifugation at 2500×g for 30 min, precipitated with polyethylene glycol (PEG):8000/NaCl solution, and resuspended in sterile phosphate-buffered saline (PBS, 137 mM NaCl, 3 mM KCl, 8 mM Na_2_HPO_4_, 1 mM KH_2_PO_4_, pH 7.4). Phage titers were scored by infecting ER2738 with serial dilutions of phage and reported as colony-forming units (CFU) [31]. The CFU assay was performed by mixing 5 µl of diluted phage sample with 45 µl of bacteria, incubation for 30 min at 37°C and then spreading on YT/AG agar plates. Colonies were counted after overnight incubation.

### Phage Assembly of pIII-fused Proteins

Recombinant phage particles (10^9^ or 10^10^ CFU) were resuspended in SDS sample buffer, boiled for 5 min, and then separated on 10% SDS-PAGE reducing gel. After transfer to an Immobilon®-P PVDF transfer membrane (Millipore, Bedford, MA) and blocking with 3% BSA in PBST, Western blotting was performed with a mouse anti-M13 pIII monoclonal antibody or a mouse anti-myc antibody, followed by a HRP-conjugated anti-mouse antibody. Proteins were visualized with PowerOpti-ECL Detection reagent (Animal Genetics lnc. Korea) and ImageQuant LAS 4000 mini (GE Health Care, Piscataway, NJ). Band densities were quantified using ImageJ software (National Institutes of Health, USA) [16].

### Phage ELISA

To determine the specific binding activity of recombinant phage, the antigen of either DR4 (10 µg/ml) or DNA mixture (1 µg/ml) diluted in PBS (100 µl/well) was coated in the 96-well clear flat bottom polystyrene microplates (Corning Costar Corp, Cambridge, MA) at 4°C overnight. After blocking the wells with 2% bovine serum albumin (BSA) in PBS, serially diluted recombinant phages (10^7^∼10^11^ CFU of hAY4 scFv-M13, 3D8 VL-M13, TAT-M13 phage particles) or VCSM13 helper phage were added, and incubated for 2 h at 25°C. The plates were washed 10 times with 200 µl of PBST (PBS containing 0.05% Tween 20) and then bound phage was detected by incubation with 50 µl of 1∶5000 diluted HRP-conjugated anti-M13 antibody (Abcam, Cambridge, MA) for 1 h at 25°C. The signal was visualized with 2,2′-azino-di-(3-ethylbenzthiazoline-6-sulfonic acid) (ABTS) working solution (10 µl 30% H_2_O_2_+10 ml ABTS substrate buffer [1 mM ABTS in 70 mM citrate phosphate buffer, pH 4.2]) and absorbance at 410 nm was measured on a VERSAmax microplate reader (Molecular Devices, Sunnyvale, CA). BSA served as the negative control antigen.

### Phage Internalization and Localization

Immunofluorescence microscopy was performed for *in situ* detection of internalized phage particles in cultured cells. Briefly, cells (5×10^4^) grown on coverslips in 24-well culture plates were washed, preincubated in serum-free TOM™ (Transfection optimized medium, Welgene) medium for 30 min at 37°C, and then treated with recombinant phage particles suspended in TOM™ medium, as specified in the Figure Legends. After 6 washes with cold PBS, the cells were washed 3 times for 10 min at 25°C with low pH glycine stripping buffer (50 mM glycine, 500 mM NaCl, pH 2.5), followed by 2 additional washes with PBS, to remove surface-bound phages. After fixation with 2% paraformaldehyde (PFA) in PBS for 10 min at 25°C, permeabilization with PERM-buffer (0.1% saponin, 0.1% sodium azide, 1% BSA in PBS) for 10 min at 25°C, and then blocking with 2% BSA in PBS for 1 h at 25°C, internalized phages were detected with mouse anti-M13 major coat protein pVIII antibody (diluted 1∶250 in 2% BSA in PBS) for 2 h at 25°C, followed by FITC-anti-mouse antibody (1∶500) or TRITC-anti-mouse antibody (1∶500) for 1 h at 25°C. After mounting the coverslips onto glass slides with VECTASHIELD (mounting medium with DAPI, Vector Laboratories, Burlingame, CA), optical confocal sections were obtained on a Zeiss LSM710 system with ZEN software (Carl Zeiss, Ltd., Welwyn Garden City, UK). In the experiment with soluble GAGs, cells were pretreated with 100 IU/ml heparin, 50 µg/ml chondroitin sulfate A, chondroitin sulfate B, chondroitin sulfate B, or dextran sulfate for 30 min at 37°C prior to addition of recombinant phage particles [28]. In the experiment with endocytosis inhibitors, cells were pretreated with CPZ (1 µg/ml), MβCD (5 mM), or Cyt-D (1 µg/ml) for 30 min at 37°C prior to addition of recombinant phage particles [15]. In the experiment with endocytosis marker, Alexa 488-TF (10 µg/ml), Alexa 488-Ctx-B (10 µg/ml), or FITC-dextran (10 µg/ml) was co-treated with recombinant phage particles. In the experiment with specific GAG lyases, cells were treated for 2 h at 37°C with 5 mIU/ml heparinase III reconstituted in buffer containing 20 mM Tris-HCl, pH 7.5, 0.1 mg/ml BSA, and 4 mM CaCl_2_ or 20 mIU/ml chondroitinase ABC in buffer containing 50 mM Tris, pH 8.0, 60 mM sodium acetate, and 0.02% BSA. Subsequently the cells were washed gently 6 times with PBS prior to addition of recombinant phage particles [28].

### Cellular Recovery and Titration of Internalized Phages

For the titration of internalized phage particles, about 1×10^6^ cells grown in maintenance medium on 60-mm culture dishes were washed, preincubated in serum-free TOM™ medium for 30 min at 37°C, and then treated with 10^10^ to 10^13^ CFU of recombinant phages suspended in TOM™ medium at 37°C for 2∼6 h, as specified in Figure Legends. After 6 washes with cold PBS, the cells were washed 3 times at 25°C for 10 min with low pH glycine stripping buffer (50 mM glycine, 500 mM NaCl, pH 2.5), followed by 2 additional washes with PBS, to remove surface-bound phages. The cells were lysed in 0.1 ml PBS by 2 cycles of freezing/thawing, and the cell lysates collected by centrifugation at 12000 rpm for 15 min at 4°C were used to infect *E.coli* XL-1 Blue cells. The output phage titer was determined by the CFU assay [32].

### RNA Interference

HeLa cells grown to about 50% confluence on a 6- or 24-well culture plate were transfected with small interfering RNA (siRNA) specific for clathrin, caveolin-1, or dynamin-2 with the WelFect-EX^TM^PLUS transfection reagent kit (Welgene, Korea) according to manufacturer protocols. After 48 h, protein levels were analyzed by Western blotting and internalized phages were detected by confocal microscopy. The mRNA sequences targeted by each siRNA were as follows [15] : clathrin siRNA, 5′-AAC CUG CGG UCU GGA GUC AAC-3′, caveolin-1siRNA, 5′-AGA CGA GCU GAG CGA GAA GCA-3′, dynamin-2 siRNA, 5′-GUG GAC CUG GUU AUC CAG GAG CUA A-3′. An unrelated siRNA with a scramble sequence of 5′-CCU ACG CCA CCA AUU UCG U-3′ was used as a control.

### Pulse-chase Experiment to Monitor Intracellular Trafficking of Internalized Phages

To track internalization of recombinant phage particles, cells (5×10^4^) grown on coverslips in 24-well culture plates were incubated with 10^12^ CFU of 3D8 VL-M13 for 2 h or 10^13 ^CFU of TAT-M13 for 30 min at 37°C, quickly washed 3 times with PBS, and then incubated at 37°C for the indicated times, as specified in Figure Legends. After washing, stripping, fixation, permeabilization, and blocking of the cells, internalized phages and subcellular organelles were stained by incubation with anti-M13 major coat protein and anti-EEA1, anti-caveolin-1, anti-calnexin, and anti-58K Golgi protein, followed by the appropriate secondary Abs conjugated to TRITC or FITC. To visualize lysosome, LysoTracker®Red DND-99 diluted in medium (1 µM) that does not require use of secondary antibody for detection, was added directly in the cell culture for 30 min at 37°C in a 5% CO_2_ incubator.

### Statistical Analysis

Data are reported as the mean ± S.E. values of at least 3 independent experiments carried out in triplicate, unless otherwise specified. Statistical significance was analyzed by the two-tailed Student's *t*-test in Sigma Plot 8.0 software (SPSS Inc.), and a *P* value of less than 0.05 was considered statistically significant.

## Results

### Production of Recombinant Filamentous M13 Phages Displaying 3D8 VL or TAT

**Figure 1:**
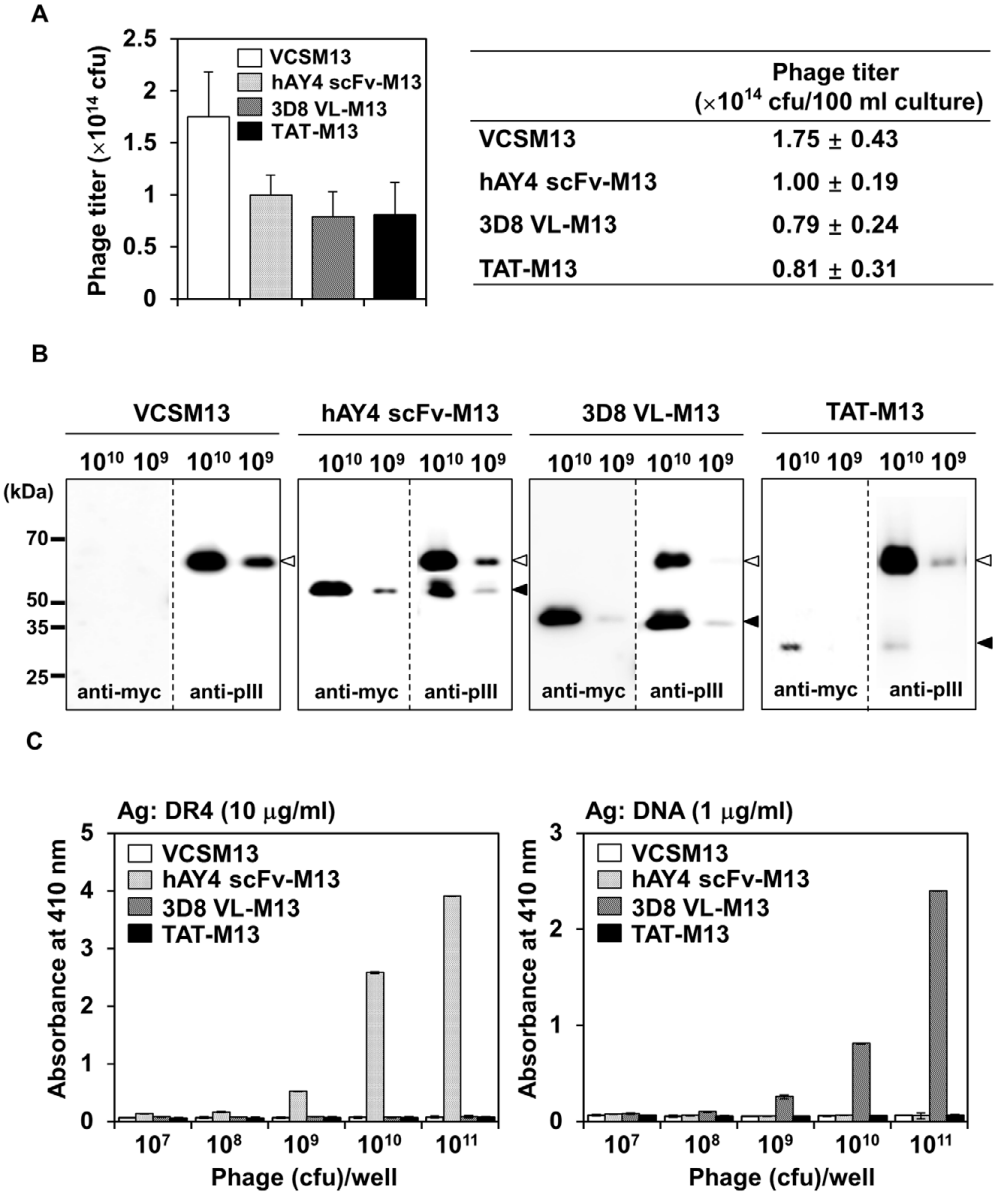
Generation of filamentous M13 phages displaying cell-penetrating 3D8 VL transbody (3D8 VL-M13) and TAT peptide (TAT-M13). As a control, anti-DR4 hAY4 scFv without cell-penetrating ability was also employed; the phage particles are designated as hAY4 scFv-M13. (**A**) Phage titers obtained from 100-ml culture supernatants of recombinant phagemid-transformed bacteria by VCSM13 helper phage superinfection under optimal culture conditions as described in the text. Phage titers were determined by CFU assay. Data represent mean ± S.E. (*error bars*) of 5 independent experiments. (**B**) Western blot analysis of display efficiency of fusion proteins (insert-pIII) (*filled arrow*) *versus* full-length pIII (*open arrow*) from VCSM13 helper phage on the recombinant M13 filamentous phage particles. Equal titers (10^9^ or 10^10^ CFU) of phage particles prepared as described in (A) were western blotted with anti-myc antibody to detect only pIII-fusion proteins (3D8 VL-pIII, and TAT-pIII, and hAY4 scFv-pIII) from the phagemid vectors or anti-M13 pIII antibody to detect both pIII-fusion proteins from the phagemid vectors and full-length pIII from the helper phage. The positions of molecular size marker are indicated. (**C**) Phage ELISA on DR4 or DNA to examine antigen-binding specificity of the recombinant phages. Various titers (10^7^∼10^11^ CFU) of recombinant phages or VCSM13 helper phage were applied to each well, precoated with the indicated antigen. Bound phages were detected with HRP-conjugated anti-M13 antibody. Data represent mean ± S.E. (*error bars*) of three independent experiments carried out in triplicate.

We used a standard filamentous M13 phagemid system to display the cell-penetrating single domain 3D8 VL transbody and TAT peptide on the phage surface by fusion to the N-terminus of truncated minor coat protein pIII (residues 230–406) [29] (Figure S1A). Anti-DR4 hAY4 scFv, which specifically binds DR4 without cellular internalization [30], was also fused to pIII as a control. The fusion phagemid DNA can be packaged into recombinant phage particles by superinfection with VCSM13 helper phage which supplies all the proteins necessary for viral assembly [29], [31]. The recombinant phage displays 2–4 copies of full-length pIII derived from the helper phage and usually 1–2 copies of the recombinant pIII fusion proteins 3D8 VL-pIII, TAT-pIII, or hAY4 scFv-pIII derived from the phagemid; the relative display ratio depends on the passenger proteins (molecular size, conformation, electric charge, etc.) and culture conditions [33]. Thus we first determined optimal display conditions for each fusion protein by determining the ratio of fusion proteins to intact pIII protein by Western blotting for recombinant phage particles prepared at various culture temperature and times. The results showed that phage surface display of fusion proteins was highest in the culture conditions of growth at 20°C for 20 h without IPTG stimulation for 3D8 VL-pIII and hAY4 scFv-pIII, but growth at 37°C for 20 h with 0.5 mM IPTG stimulation for TAT-pIII (Figure S1B). Subsequently, we prepared the recombinant phages under the optimal conditions defined for each fusion protein. Under optimal display culture conditions, the titers of recombinant phage progeny obtained from 100 ml culture supernatants ranged from 0.8 to 1.0×10^14^ CFU (Fig. 1A), comparable to other pIII fusion proteins [29], [31]. To determine the relative display level of fusion proteins, an equivalent titer of recombinant phages (10^10^ or 10^9^ CFU) was analyzed by Western blotting using anti-myc antibody to detect only the pIII-fusion proteins or anti-pIII antibody to detect both pIII-fusion and intact full-length pIII proteins. 3D8 VL-pIII and hAY4 scFv-pIII were readily detected at a titer as low as 10^9^ CFU, whereas TAT-pIII was weakly detected at 10^10^ CFU, but was negligible at 10^9^ CFU (Fig. 1B). Densitometry analysis of the Western blots revealed that the ratio of pIII-fused to intact full-length pIII was approximately 1∶2.5 for hAY4-M13, 1.5∶1 for 3D8 VL-M13, and 1∶20 for TAT-M13. Thus phage-surface display of 3D8 VL is more efficient than TAT peptide by ∼30-fold.

To monitor whether phage surface-displayed 3D8 VL and hAY4 scFv retain specific antigen-binding capability, we performed phage ELISA against the cognate antigen, i.e., DNA for 3D8 VL [12], [13] and DR4 for hAY4 scFv [30]. 3D8 VL-M13 and hAY4-M13 phages only showed titer-dependent specific binding to DNA and DR4 (Fig. 1C). The helper and TAT-M13 phages bound to neither DR4 nor DNA. Thus 3D8 VL and hAY4 fused with pIII is functionally displayed and is accessible for antigen interaction.

### 3D8 VL-M13 and TAT-M13 Phages are Internalized by an Energy-dependent Mechanism and can be Rescued from within the Cells in the Infective form in Proportion to the Input Titer

**Figure 2:**
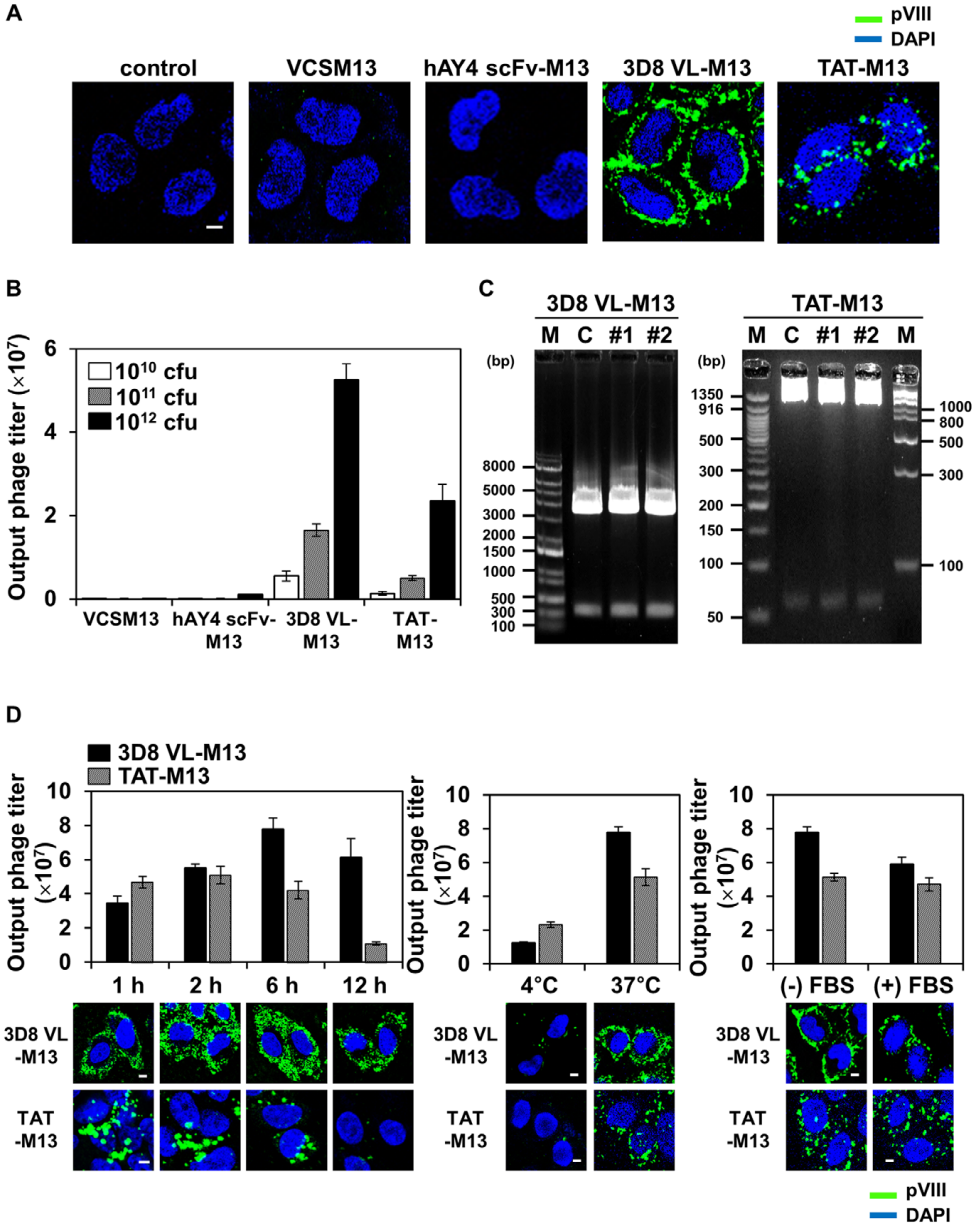
3D8 VL-M13 and TAT-M13 phages penetrate living cells in an energy-dependent manner and in the presence of serum proteins, and can be rescued in their infective form in proportion to the input titer. Unless otherwise specified, HeLa cells (1×10^6^ cells) in serum-free medium were treated at 37°C with 10^12^ CFU of VCSM13, 3D8 VL-M13, or hAY4 scFv-M13 for 6 h or 10^13^ CFU of TAT-M13 for 2 h. (**A**) Internalization and subcellular localization of phage particles in HeLa cells, untreated (‘control’) or treated with VCSM13 helper phage or the indicated recombinant phages and analyzed by confocal immunofluorescence microscopy. Internalized phages were detected by primary anti-pVIII antibody and secondary FITC-anti-mouse antibody. Images show the merging of phages (*green*) and DAPI-stained nuclei (*blue*) at the centered single confocal section. (**B**) Effect of input phage titer on the recovery of internalized recombinant phages. HeLa cells were incubated with 10^10^, 10^11^, or 10^12^ CFU of phages (input phage titer), thoroughly washed with low pH glycine buffer to remove surface bound phages, and lysed. Bacteria were infected with the cell lysates to determine output phage titer by CFU assay. Data represent mean ± S.E. of 3 independent experiments. (**C**) Plasmid DNA analysis from the recovered phage particles. Cell lysates prepared as described in (B) were transformed into bacteria and then plasmid DNA was extracted from randomly chosen two colonies (*#1*, *#2*). The recovered plasmid DNA was digested with *Sfi*I to excise the DNA insert [3D8 VL (366 bp) or TAT (62 bp)] prior to agarose gel electrophoresis and visualization by ethidium bromide staining. ‘C’ indicates the original phagemid vector carrying the 3D8 VL or TAT gene. ‘M’, DNA size marker. (**D**) Effects of incubation time, temperature, and presence of serum proteins on internalization of 3D8 VL-M13 and TAT-M13 phages. HeLa cells were incubated with 3D8 VL-M13 or TAT-M13 for the indicated periods (*left panels*), at 4°C or 37°C (*middle panels*), or at 37°C in the presence or absence of 10% fetal bovine serum (*right panels*). Internalized phages were quantified by the CFU assay and represented as mean ± S.E. of 2 independent experiments as described in (B) and visualized by confocal immunofluorescence microscopy as described in (A). In (A) and (D), image magnification, ×400; scale bar, 5 µm.

To determine whether phage surface-displayed 3D8 VL and TAT can facilitate cellular internalization of the recombinant phages, we incubated human cervical carcinoma HeLa cells (∼1×10^6^ cells) at 37°C with 10^12^ CFU of VCSM13, hAY4 scFv-M13, and 3D8 VL-M13 phages for 6 h or 10^13^ CFU of TAT-M13 phages for 2 h to allow internalization. The 10-fold higher titer of TAT-M13 was employed considering the much lower display level of TAT than that of 3D8 VL on the phage surface (Fig. 1B). After stringent washing to remove cell surface-bound phages [19], [21], internalized phages were detected by confocal immunofluorescence microscopy with antibody recognizing the major coat protein pVIII of M13. As expected, VCSM13 helper phage and hAY4 scFv-M13 were not detected in the interior of cells. For 3D8 VL-M13 and TAT-M13 phages, however, most cells uniformly exhibited intracellular fluorescence as evidenced by confocal section analysis (Fig. 2A and Figure S2B), demonstrating efficient cellular internalization of the recombinant phages. Interestingly, 3D8 VL-M13 was detected as a scattered, fine granular pattern throughout the cytosol; little was detected in the nucleus. TAT-M13 was also detected in punctate, vesicle-like compartments, but was distributed around and inside the nucleus (Fig. 2A), consistent with a previous report of TAT-mediated M13 phage internalization [24]. 3D8 VL-M13 and TAT-M13 also penetrated A375-SM melanoma and MDA-MB-231 breast cancer cells with a similar pattern of immunostaining and cellular distribution as in HeLa cells (Figure S2A), suggesting that the cell-penetrating ability of 3D8 VL-M13 and TAT-M13 is not cell-type specific, as are the soluble 3D8 VL transbody and TAT peptide [13], [14], [15]. The internalization of 3D8 VL-M13 and TAT-M13 phages did not cause any noticeable cytotoxicity for HeLa cells during 24 h incubation (Figure S3). To our knowledge, this study is the first to demonstrate cellular internalization of phage mediated by a cell-penetrating antibody.

Previous studies have shown that phages rescued from within cells after phage internalization can infect bacteria [19], [32]. After incubation of HeLa cells with various titers of the recombinant phages (input titer) and stringent washing, the cell lysates were used to infect bacteria to recover the internalized phages and quantify output phage titers. Indeed, 3D8 VL-M13 and TAT-M13 recovered from within the cells retained its infectivity showing output phage titer proportional to the dose of input phages (Fig. 2B). Internalization efficiency (the ratio of output to input phage) of 3D8 VL-M13 (0.005∼0.01%) was about 10-fold higher than that of TAT-M13 (0.001∼0.005%), consistent with the greater intracellular fluorescence signal of 3D8 VL-M13 versus TAT-M13 (Fig. 2A and 2D). Plasmid DNA extracted from bacteria after infection with cell lysates containing 3D8 VL-M13 and TAT-M13 particles was the original phagemid containing the 3D8 VL- or TAT-encoding DNA insert (Fig. 2C). These results suggest that the phage internalization is titer-dependent and that at least some portion of internalized phages remain intact after cellular internalization.

Cellular internalization of 3D8 VL-M13 and TAT-M13 was further characterized by incubation of the cells at 37°C with a fixed titer of phages for 1, 2, 6, and 12 h; incubation at 4°C; or incubation at 37°C with or without 10% FBS. The output titer of 3D8 VL-M13 increased gradually with incubation time up to 6 h; at 12 h the titer slightly reduced but was still high (Fig. 2D). In contrast, the output titer of TAT-M13 plateaued at 1∼2 h incubation and then decreased after 6 h of incubation. Cellular internalization of 3D8 VL- and TAT-M13 phages was greatly impaired at 4°C incubation, but only marginally decreased at 37°C in the presence of 10% FBS (Fig. 2D), indicating that the phage internalization involves energy-dependent endocytotic processes without significant interference by serum components, as observed for soluble counterpart proteins [7], [13] and filamentous fd internalized by surface display of Her2-specific scFv [19].

### Endocytotic Mechanisms Underlying 3D8 VL-M13 and TAT-M13 Internalization

**Figure 3:**
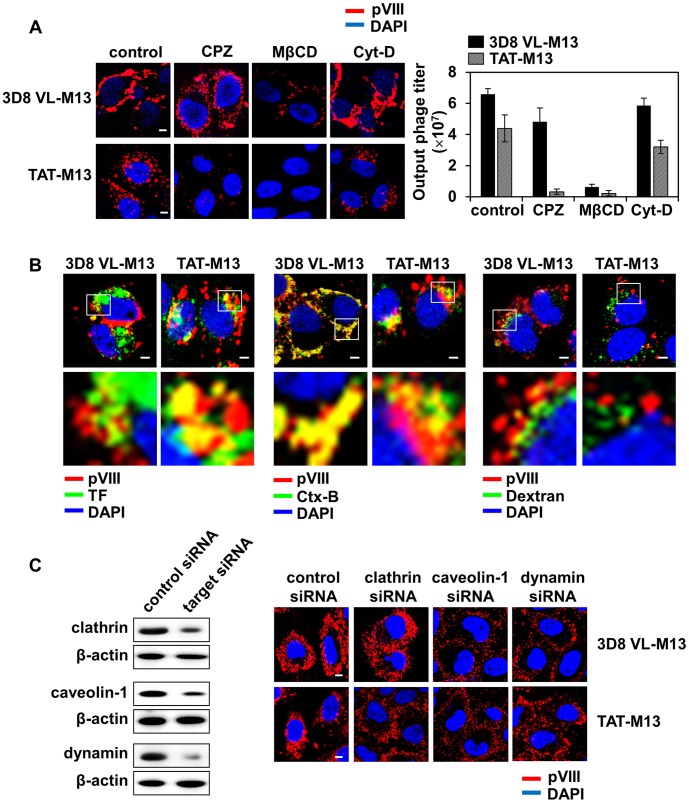
3D8 VL-M13 is internalized by caveolae-mediated endocytosis, whereas TAT-M13 by clathrin- and caveolae-mediated endocytosis. Unless otherwise specified, HeLa cells (1×10^6^ cells) in serum-free medium were treated at 37°C with 10^12^ CFU of 3D8 VL-M13 for 6 h or 10^13^ CFU of TAT-M13 for 2 h. (**A**) Effect of pre-treatment of specific endocytosis inhibitors on the internalization of 3D8 VL-M13 or TAT-M13. HeLa cells were pre-treated with CPZ (1 µg/ml), MβCD (5 mM), or Cyt-D (1 µg/ml) for 30 min and then incubated with 3D8 VL-M13 or TAT-M13. Internalized phages were visualized by confocal immunofluorescence microscopy using primary anti-pVIII antibody and secondary TRITC-anti-mouse antibody or quantified by the CFU assay and represented as mean ± S.E. (*error bars*) of 3 independent experiments. Images show the merging of phages (*red*) and DAPI-stained nuclei (*blue*) at the centered single confocal section. (**B**) Co-localization of internalized 3D8 VL-M13 or TAT-M13 with intracellular endocytosis markers. Cells were co-treated for 2 h with the recombinant phages and endocytosis markers, 10 µg/ml Alexa 488-transferrin (TF, *green*), Alexa 488-choleratoxin-B (Ctx-B, green), or FITC-dextran (Dextran, green), and then analyzed by confocal microscopy after staining for internalized phages (TRITC, *red*) as described in (A). The lower panels show enlarged images of the boxed region in the upper panels. (**C**) Knockdown of clathrin, caveolin-1, or dynamin by specific siRNA, monitored by Western blotting (*left panels*), and the effects on internalization of 3D8 VL-M13 and TAT-M13 (*right panels*). HeLa cells were transfected with the indicated siRNA for 48 h and then incubated with 3D8 VL-M13 and TAT-M13 prior to phage visualization by confocal immunofluorescence microscopy as described in (A). ‘Control siRNA’ means a scrambled siRNA used as a control. In (A–C), image magnification, ×400 and scale bar, 5 µm.

To elucidate the internalization mechanism of 3D8 VL-M13 and TAT-M13, we first tested pharmacological inhibitors of the major physiological endocytotic pathways, including CPZ (inhibitor of clathrin-mediated endocytosis), MβCD (inhibitor of caveolae-mediated endocytosis), and Cyt-D (inhibitor of macropinocytosis) [1], [15]. MβCD, but neither CPZ nor Cyt-D, significantly reduced cellular internalization and output phage titer of 3D8 VL-M13 (Fig. 3A). TAT-M13 internalization was almost completely abolished by CPZ and MβCD, but only slightly inhibited by Cyt-D. To further clarify the specific endocytosis pathways, 3D8 VL-M13 and TAT-M13 phages were co-stained with FITC-labeled intracellular trafficking markers for the endocytosis pathways, including TF for clathrin-mediated endocytosis, Ctx-B for caveolae-mediated endocytosis, and dextran for macropinocytosis [1], [15]. 3D8 VL-M13-containing compartments co-localized mainly with Ctx-B, but rarely with TF or dextran, whereas TAT-M13-containing vesicles predominantly co-localized with TF and Ctx-B, but not with dextran (Fig. 3B).

Knockdown of genes involved in clathrin- or caveolae-mediated endocytosis was performed using siRNA techniques. Clathrin is required for clustering of ligand-receptor complexes in coated pits prior to invagination and pinching off of the plasma membrane to form intracellular clathrin-coated vesicles [1], [15]. Caveolin-1 is a principal structural component of the cholesterol/sphingolipid-enriched plasma membrane caveolae [1], [15]. Dynamin, a kind of GTPase, mediates clathrin- and caveolae-mediated endocytosis by forming a spiral around the neck of newly formed invaginating vesicles and then pinching off the vesicles from the parent membrane [1], [15]. After 48 h of siRNA transfection, expression of clathrin, caveolin-1, and dynamin was reduced by 60∼90% (Fig. 3C). Depletion of caveolin-1 and dynamin, but not clathrin, significantly impaired 3D8 VL-M13 internalization and trapped the phages on the plasma membrane (Fig. 3C). TAT-M13 was stuck around the plasma membrane without further trafficking to the perinuclear regions by knockdown of clathrin, caveolin-1, and dynamin (Fig. 3C). Collectively, the above biochemical and knock-down results indicate that cellular internalization of 3D8 VL-M13 occurs primarily through dynamin-dependent caveolae-mediated endocytosis, similar to soluble 3D8 VL protein [13], while TAT-M13 is internalized into cells through simultaneous uses of the two endocytotic pathways of clathrin- and caveolae-mediated endocytosis.

### 3D8 VL-M13 and TAT-M13 Phages Utilize HSPG and CSPG, Respectively, as Primary Cell Surface Receptors for the Cellular Internalization

**Figure 4:**
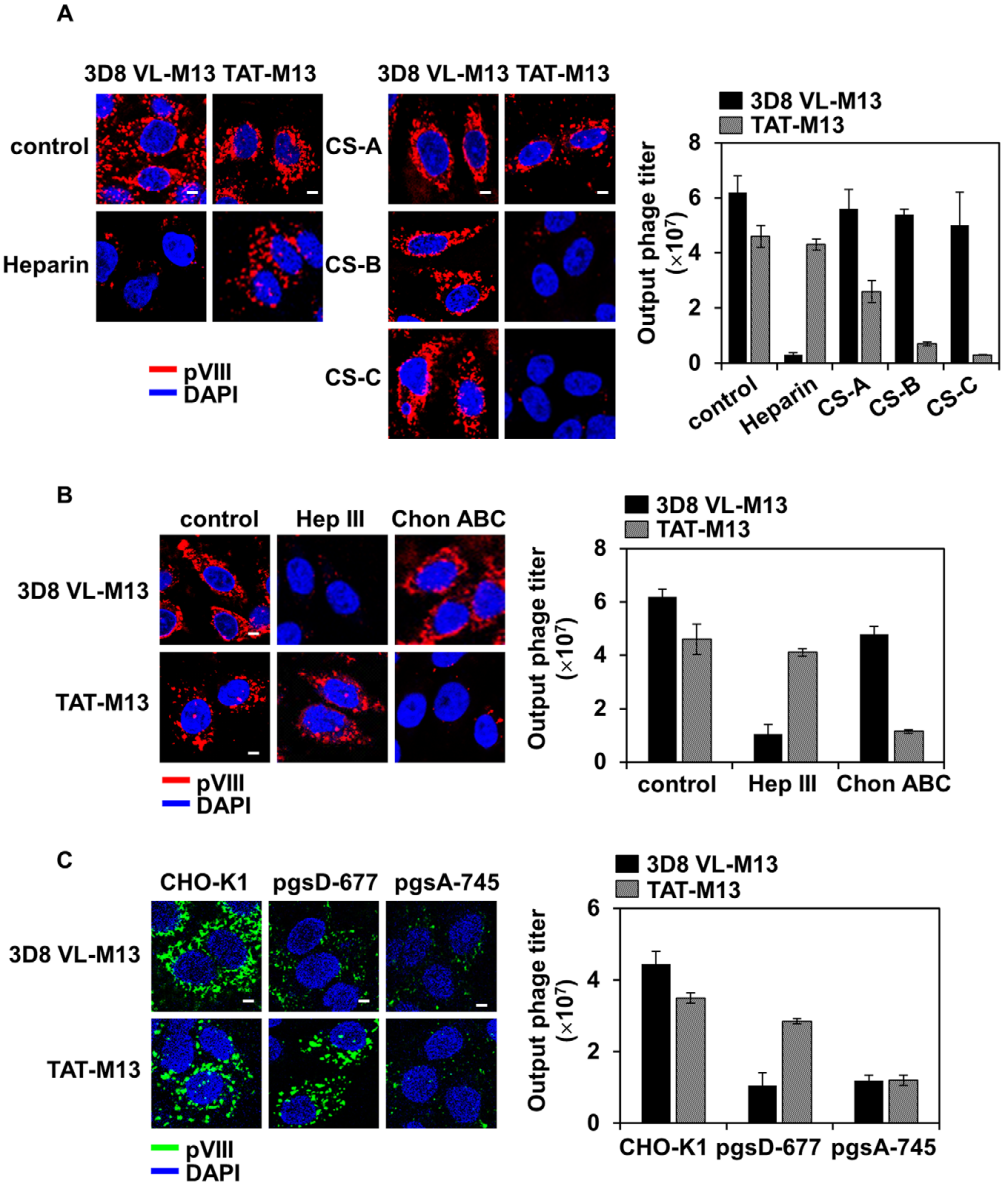
3D8 VL-M13 and TAT-M13 mainly interact with HSPG and CSPG, respectively, as a cell surface receptor for the cellular internalization. In all experiments, wild-type CHO-K1 and the mutant cells (1×10^6^ cells) in serum-free medium were treated at 37°C with 10^12^ CFU of 3D8 VL-M13 for 6 h or 10^13^ CFU of TAT-M13 for 2 h. (**A**) Effects of the presence of soluble GAGs on internalization of 3D8 VL-M13 and TAT-M13. CHO-K1 cells were pre-treated with 100 IU/ml of heparin, or 50 µg/ml of CS-A, CS-B, and CS-C for 30 min and then incubated with 3D8 VL-M13 or TAT-M13. (**B**) Effects of treatment of cells with GAG lyases on the internalization of 3D8 VL-M13 and TAT-M13. CHO-K1 cells were pre-treated with 5 mIU/ml of heparinase III (Hep III) or 20 mIU/ml of chondroitinase ABC (Chon ABC) for 2 h at 37°C and then incubated with 3D8 VL-M13 or TAT-M13. (**C**) Internalization of 3D8 VL-M13 and TAT-M13 into CHO-K1 mutant cells genetically defective in GAG biosynthesis. Wild-type CHO-K1, HS-deficient pgsD-677 (no HS, 3 times more CS), or HS/CS-deficient pgsA-745 (no proteoglycans) cells were incubated with 3D8 VL-M13 or TAT-M13. In (A–C), internalized phages were visualized by confocal immunofluorescence microscopy using primary anti-pVIII antibody and secondary TRITC-anti-mouse antibody (A and B, *red*) or FITC-anti-mouse antibody (C, *green*) or quantified by the CFU assay and represented as mean ± S.E. (*error bars*) of three independent experiments. Image magnification, ×400; scale bar, 5 µm.

Previous reports have shown that cellular uptake of 3D8 VL transbody and TAT peptide is initiated by non-specific electrostatic interactions with negatively charged GAGs, such as HS and CS, linked as side chains to cell surface core proteins to form proteoglycans HSPG and CSPG [7], [13], [28]. To determine which GAG(s) is responsible for internalization of 3D8 VL-M13 and TAT-M13 phages, we first examined the effects of soluble GAGs, such as heparin, CS-A, CS-B, and CS-C, on phage internalization into hamster CHO-K1 cells. The presence of exogenous heparin, a close structural homologue of HS, significantly blocked internalization and intracellular recovery of 3D8 VL-M13, but not TAT-M13 (Fig. 4A). Exogenous CSs did not impede 3D8 VL-M13 internalization and recovery. In contrast, TAT-M13 internalization and rescue were reduced dramatically by CS-B and CS-C, but only marginally by CS-A (Fig. 4A). Next, the effects of enzymatic removal of cell surface GAGs were examined by treating CHO-K1 cells with specific GAG lyases, such as heparinase III to remove endogenous HS and chondroitinase ABC to remove endogenous CSs (CS-A, -B, -C) [28] prior to phage incubation. Internalization and recovery of 3D8 VL-M13 was severely impaired in cells treated with heparinase III, but not chondroitinase ABC (Fig. 4B). In contrast, TAT-M13 was efficiently internalized into heparinase III-treated cells, but not chondroitinase ABC-treated cells (Fig. 4B). To provide genetic evidence of the role of HS and CS GAGs in phage internalization, we tested mutant derivatives of CHO-K1 cells: HS-deficient pgsD-677 and HS/CS-deficient pgsA-745 cells [27], [28]. 3D8 VL-M13 internalization was severely impaired in pgsD-677 and pgsA-745 cells, more dramatically in pgsA-745 cells, which lack HS and CS GAGs on the cell surface (Fig. 4C and Figure S4). In contrast, TAT-M13 internalization was significantly reduced in pgsA-745 cells, but marginally inhibited in pgsD-677 cells. Overall, these results provide genetic and biochemical evidences that cell surface HSPGs are the major cellular receptors for cellular internalization of 3D8 VL-M13 phage, while CSPGs, particularly with CS-B and CS-C, mediate TAT-M13 internalization.

### Intracellular Trafficking of Internalized 3D8 VL-M13 and TAT-M13 Phages

**Figure 5:**
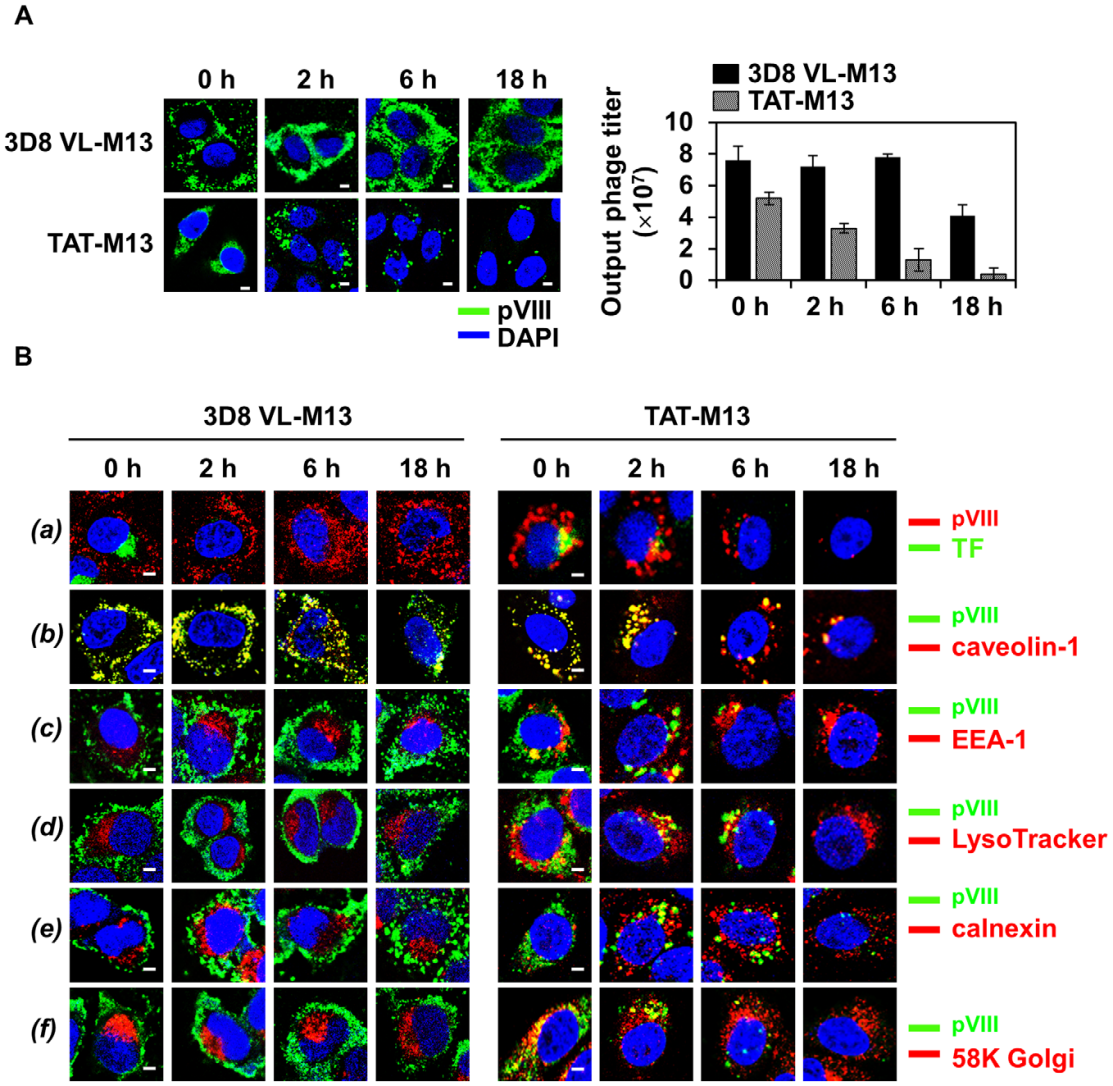
Internalized 3D8 VL-M13 phage routes to the cytosol and remains stable without further trafficking to other subcellular compartments, whereas TAT-M13 phage is routed to other subcellular compartments before rapid degradation in the lysosome. In the following pulse-chase experiments, HeLa cells (1×10^6^ cells) in serum-free medium were treated at 37°C with 10^12^ CFU of 3D8 VL-M13 for 2 h or 10^13^ CFU of TAT-M13 for 30 min. Then surface bound phages were removed by multiple washes with low pH glycine buffer and then internalized phages were chased at 0, 2, 6, and 18 h. (**A**) Time-course intracellular localization of internalized phages was visualized by confocal immunofluorescence microscopy or time-course output phages were quantified by the CFU assay and represented as mean ± S.E. (*error bars*) of three independent experiments. (**B**) Time-course intracellular trafficking of internalized phages monitored by co-localization with transferrin (TF, *a*), caveolin-1 (*b*), early endosome marker EEA-1 (*c*), late endosome/lysosome tracker LysoTracker (*d*), ER marker calnexin (*e*), or Golgi marker 58K Golgi protein (*f*), as visualized by confocal immunofluorescence microscopy. In (A) and (B), internalized phages were visualized by confocal immunofluorescence microscopy with primary anti-pVIII antibody and secondary FITC-anti-mouse antibody (A and B, *a*, *green*) or TRITC-anti-mouse antibody (B, *b-f*, *green*). In (A) and (B), magnification, ×400; scale bar, 5 µm.

To determine whether internalized phages are degraded quickly or persisted for a long period inside of the cells, pulse-chase experiments were performed. HeLa cells were incubated with 3D8 VL-M13 for 2 h or TAT-M13 for 30 min and stringently washed to remove extracellular-bound phages, and then time-course intracellular amounts of pulsed phages were estimated qualitatively by fluorescence microscopy and quantitatively by CFU assay. The shorter incubation periods than the time showing maximal internalization level of the phages, i.e., ∼6 h for 3D8 VL-M13 and 1∼2 h for TAT-M13 (Fig. 2D), were employed to monitor the intracellular stability and trafficking from the earlier stage of internalization. 3D8 VL-M13 phages were recovered at similar levels between 0 and 6 h and at ∼2-fold reduction after 18 h, but substantial amounts of phage remained in the cytosol as shown by confocal microscopy even at 18 h of internalization (Fig. 5A). In contrast, the recovery titer of TAT-M13 rapidly diminished to ∼60% of pulsed phages at 2 h, eventually diminishing to less than ∼10% at 6 h, and then below 4% at 18 h, consistent with immunofluorescence detection by confocal microscopy (Fig. 5A). These results suggest that internalized 3D8 VL-M13 is relatively stable within the cells even 18 h after internalization, but TAT-M13 is quickly degraded after 2 h of internalization.

To understand the above distinct intracellular stability, time-course intracellular routes of pulsed 3D8 VL-M13 and TAT-M13 phages were further followed by costaining with endocytotic vesicle markers and subcellular organelle markers of the early endosome, lysosome, Golgi apparatus, and endoplasmic reticulum (ER) compartments [15]. 3D8 VL-M13 did not colocalize with co-treated TF, the clathrin-mediated endocytosis marker, which rapidly disappeared within 2 h of internalization (Fig. 5B, *a* and Figure S5), consistent with their different endocytosis routes (Fig. 3B). However, a portion of vesicles containing both TAT-M13 and TF was observed immediately after internalization and almost disappeared at 2 h, implying rapid degradation of TAT-M13 internalized by clathrin-mediated endocytosis. In the case of caveosome trafficking, 3D8 VL-M13 and TAT-M13 co-localized with caveolin-1 immediately after internalization and subsequently for 2 h, consistent with earlier results (Fig. 3B). Thereafter, 3D8 VL-M13 signal was significantly separated from the caveolin-1 signal at 6 h and more so at 18 h, diffusely distributing throughout the cytosol, suggesting that 3D8 VL-M13 is released from caveosomes after 2 h of internalization (Fig. 5B, *b* and Figure S5). 3D8 VL-M13-containing caveosomes were not transported to the early endosome, lysosome, ER and Golgi up to 18 h before release of 3D8 VL-M13 into cytosol (Fig. 5B, *c–f* and Figure S5). Thus internalized 3D8 VL-M13 diffused directly into the cytosol from the caveosomes without crossing into other subcellular organelles and/or intermediate vesicles, thereby persisting in the cytosol without significant degradation. In contrast, TAT-M13-containing caveosomes were substantially observed up to 2 h, a longer persistence than TAT-M13-containing vesicles taken up by clathrin-mediated endocytosis, but substantially disappeared at 6 h when caveolin-1 signaling separated. This suggests that TAT-M13-containing caveosomes are somewhat more stable than clathrin-coated vesicles but underwent degradation after 2 h of internalization. At 18 h, caveolin-1 signals were significantly reduced, probably because caveosomes merge with the late endosome and lysosome and are completely degraded there [34]. Upon internalization, a portion of TAT-M13-containing vesicles also routed to the endosome, Golgi apparatus, ER, and lysosome compartments between 0 to 6 h and then predominantly to the lysosome at 6 h before disappearing by 18 h after internalization (Fig. 5B). This observation indicates that TAT-M13-containing vesicles are transported to several subcellular compartments, but are eventually destined for lysosomal degradation, explaining the rapid decrease in output phage titers immediately after internalization (Fig. 5B).

## Discussion

We have shown that cellular internalization of filamentous M13 bacteriophage mediated by surface-displayed cell-penetrating 3D8 VL transbody and TAT peptide occurs through physiologically relevant, energy-dependent endocytotic pathways via specific interactions with cell surface receptors. 3D8 VL-M13 phage was internalized by caveolae-mediated endocytosis via HSPG as the main cell surface receptor, was predominantly localized in the cytosol, and remained stable for more than 18 h after internalization without further trafficking to other subcellular compartments. Meanwhile, TAT-M13 phage was internalized by clathrin- and caveolae-mediated endocytosis with CSPG as the primary cell surface receptor and was routed to several subcellular compartments before degradation in the lysosome even after 2 h of internalization.

Internalized 3D8 VL-M13 and TAT-M13 phages can be rescued in their infective form by cell lysis, allowing quantification by infection into bacteria (Fig. 2), like filamentous phages internalized by receptor-specific scFv antibody [19], [21]. The internalizing efficiency of the recombinant phages was proportional to the input phage titer with 3D8 VL-M13 providing higher yield (0.005∼0.01%) than TAT-M13 (0.001∼0.005%). Phage surface display of 3D8 VL was about 30-fold higher than that of TAT peptide (Fig. 1), which accounts for the more efficient internalization of 3D8 VL-M13 (Fig. 2). We speculate that the positively charged TAT peptide may interfere with the assembly of recombinant TAT-pIII fused protein onto the phage tip [33]. 3D8 VL transbody has a typical immunoglobulin fold with high solubility in bacterial expression [12], [35] and its cell-penetrating ability is conferred by its unique cationic patch composed of Arg27f, Arg29, and Lys30 on CDR1 [13], [14], [35]. Thus 3D8 VL with its cationic-patch embedded in the stable folded structure seems to be superior in expression and assembly in the pIII-fused form, versus TAT with cationic residues in a short randomly structured peptide. Therefore, the 3D8 VL transbody is a more suitable carrier for phage internalization than cationic CPPs, including TAT.

Enzymatic or genetic removal of cell surface-expressed HSPG and/or CSPG and competition assays with soluble GAGs demonstrated that 3D8 VL-M13 and TAT-M13 are internalized via negatively charged sulfated proteoglycans as cell surface receptors (Fig. 4). This notion has been supported by their non-specific uptake into mammalian cells. GAGs HS and CS can serve as internalizing receptors for many positively charged molecules, microbes, viruses, and parasites [2], [6]. Like 3D8 VL-M13, some proteins and animal viruses that bind HS on HSPG are internalized via caveolae-mediated endocytosis [23], [36], [37]. A large TAT-coated nanoparticle has been reported to be internalized by simultaneous use of clathrin- and caveolae-mediated endocytosis [38], like TAT-M13. However, many proteins and CPPs are internalized by clathrin-mediated endocytosis via HSPG and caveolae-mediated endocytosis via CSPG [2], [39]. Thus, rather than the type of cell surface receptors, the cell-penetrating moiety itself seems to determine the choice of endocytotic pathway.

Surprisingly 3D8 VL-M13 and TAT-M13 phages showed distinct specificity for their cell surface receptors; 3D8 VL-M13 internalizes through interaction with HSPG, but not CSPG, whereas TAT-M13 internalizes through CSPG rather than HSPG interactions. HSPG-mediated internalization of 3D8 VL-M13 is consistent with that of soluble 3D8 VL [13], [14]. However, TAT-M13 internalization through CSPG is rather intriguing given that soluble TAT and TAT-fused proteins have been reported to preferentially interact with HSPG over CSPG as cellular internalizing receptors [7], [28]. GAGs, including HS and CS, are polysaccharides composed of 20–150 repeating disaccharide units differing in the hesosamine unit (D-galactosamine or D-glucosamine), uronic acid (D-glucuronic acid or D-iduronic acid) and the number and position of sulfate groups [6]. HS differs from CS in saccharide composition and sulfation number and position on the GAG backbone, the details of which are ill defined due to their diversity in chain length, domain structure and sulfation level [6]. Nonetheless, our data suggest that the receptor-specificity for cellular internalization of 3D8 VL-M13 and TAT-M13 is determined not by non-specific electrostatic interactions, but by the fine structure of the GAG backbone, as characterized by saccharide composition and negative charge density and position. This argument is supported by a previous finding that phage-displayed anti-HS antibody with highly basic CDRs recognized specific patterns of sulfation and sugar conformation of HSPG [40]. Accordingly, although 3D8 VL and TAT share a cell-penetrating motif of clustered cationic amino acids (Lys and Arg) whose positive charges interact with the negatively charged carboxyl and sulfate groups of GAGs, differences in the arrangement of the basic amino acids may distinguish the variable positioning and density of the anionic groups in the GAG partner. Similarly, soluble TAT and phage surface-displayed TAT by fusion with pIII might have a different conformation, resulting in different preferential interactions with CSPG and HSPG.

Internalization of recombinant filamentous phages of 3D8 VL- and TAT-M13 via caveosomes and/or clathrin-coated vesicles is somewhat surprising because the typical size of vesicles (∼50–200 nm in diameter) seems too small to contain the much bigger phages (900 nm long and 10 nm wide). Confocal fluorescence microscopy of 3D8 VL-M13 and TAT-M13 phages showed staining in fine granular vesicles, indicating that the phages are trapped in compact form in order to be contained in the small vesicles. Large particles, such as bacteria and animal viruses, are taken up by phagocytosis, a process restricted to a few innate immune cells, such as macrophages and neutrophils [2], excluding it from the endocytosis mechanisms of non-phagocytic epithelial cells. Macropinocytosis, an actin-dependent endocytotic mechanism that involves internalization of relatively large (>1 µm) patches of membrane, was not involved in 3D8 VL-M13 and TAT-M13 internalization (Fig. 3). Alhough clathrin-mediated endocytosis has a strict cargo size limit of around 200 nm [41], caveolae can internalize large molecular complexes, including particles up to 500 nm [41], and may serve as a portal for certain very large particles, such as SV40 virus and bacteria [42]. Thus, it seems that association of the recombinant phages with HSPG or CSPG on the cell surface recruits additional internalization machinery to the caveolae and/or clathrin-coated vesicles to accommodate the large particles.

Our pulse-chase experiment to follow the intracellular route and eventual fate of internalized phages indicated that 3D8 VL-M13 phages internalized *via* caveolae-mediated endocytosis bypassed further endosome sorting and lysosomal degradation, but were released directly into the cytosol from the caveosome and remained in the infective form without significant degradation, as seen with soluble 3D8 VL [13], [14], [15]. Thus, 3D8 VL-mediated cell penetration of M13 phage consists of 3 steps: 1) binding to cell surface HSPG, 2) internalization by caveolae-mediated endocytosis, and 3) release into the cytoplasm. Internalized 3D8 VL-M13 remained intact and viable for more than 18 h without significant degradation in the cytosolic environment as evidenced by the high phage recovery (Fig. 5). This suggests that the cytosolic phages are rather resistant to cellular proteases and the proteasome pathway. However, the molecular mechanism by which 3D8 VL-M13 escapes from caveosomes into the cytosol, while maintaining infectivity, is not clear. We can assume that 3D8 VL with its multiple basic residues somehow disrupts caveosomes before further trafficking into other compartments and/or deprives caveosomes of the proper signals required for fusion with other cellular compartments, the molecular basis of which remains to be determined.

In contrast, TAT-M13-containing vesicles were routed to various intracellular compartments, such as early endosomes, Golgi apparatus, and ER upon internalization, and then sorted to acidified lysosomes for degradation within 2 h of internalization, concomitant with its gradual disappearance within cells (Fig. 5). Thus it seems that TAT-M13 phages remain trapped within vesicles without cytosolic release and undergo rapid degradation in the lysosome, like internalized M13 phages with surface-displayed RGD peptide [18]. This is consistent with a previous report that endosomal vesicles formed by cellular uptake of TAT are transported into Golgi apparatus and ER before degradation by merging with the lysosome [3]. Our results indicate that 3D8 VL-M13 and TAT-M13 phages follow the intracellular trafficking and eventual fate of the cellular internalizing moiety 3D8 VL transbody and TAT peptide, respectively. Thus, the carrier for cellular internalization rather than its cargo might be the determining parameter for the endocytosis routes, intracellular destination, and eventually intracellular stability of the recombinant phage particles. It is noteworthy that TAT-M13-containing caveosomes persisted longer than TAT-M13 vesicles taken up by clathrin-mediated endocytosis (Fig. 5B). Caveosomes are non-degradative endosomal compartments with neutral pH, and their intracellular trafficking is generally slower than that of the noncaveolar pathway [39], which might explain the longer stability period of TAT-M13-containing caveosomes.

### Conclusion

We have described the detailed internalization mechanism and intracellular trafficking and stability of 3D8 VL-M13 and TAT-M13 phages, which may aid in understanding the cellular entry of bacteriophages by different cell-penetrating moieties. Comparative studies revealed that the 3D8 VL transbody and TAT peptide exhibit distinct attributes as carriers of the phage cargo for cellular uptake. We showed that 3D8 VL is displayed more efficiently than TAT and mediates more efficient phage internalization. The main limitation of TAT-mediated phage internalization is sequestration of the internalized phages within endocytotic vesicles and eventual lysosomal degradation before endosomal escape, as seen with TAT-tagged cargos such as proteins and nanoparticles [3]. However, 3D8 VL delivers the phage to cytosol, avoiding the journey to the lysosome. Efficient internalization and cytosolic localization of 3D8 VL transbody-displayed phages will provide a useful tool for intracellular delivery of polar macromolecules such as proteins, peptides, and siRNAs, which require cytosolic localization.

## Supporting Information

Figure S1 **(A)** Schematic diagram of pDR-D1-based phagemid vectors constructed for phage-surface display of 3D8 VL transbody, TAT peptide, and hAY4 scFv by fusion to the N-terminus of truncated minor coat protein pIII (residues 230–406). See text for details. **(B)** Western blotting of fusion protein display efficiency (insert-pIII) (*filled arrow*) *versus* full-length pIII (*open arrow*) from the VCSM13 helper phage on M13 filamentous phage particles, obtained from bacterial cultures at the indicated expression temperature and time. Phage particles were prepared by infecting phagemid-transformed ER2738 cells with the helper phage, adding 0.5 mM IPTG (TAT-M13) or 0 mM IPTG (3D8 VL-M13, hAY4 scFv-M13) and then incubating at 20°C, 30°C, or 37°C for 20 or 40 h. An equal titer (10^10^ CFU) of phage particles was used for Western blotting with anti-M13 pIII antibody for the detection of both pIII-fusion proteins (3D8 VL-pIII, and TAT-pIII, and hAY4 scFv-pIII) from the phagemid vectors and full-length pIII from the helper phage. The calculated molecular mass based on the amino acid sequence is ∼45.5 kDa for hAY4-pIII, ∼33.0 kDa for 3D8 VL-pIII, and 22.1 kDa for TAT-pIII fusion proteins and ∼65 kDa for full-length pIII protein from helper phage.
(TIF)

Figure S2 3D8 VL-M13 and TAT-M13 phages internalize into living cells. **(A)** Internalization and subcellular localization of recombinant phages in A375-SM and MDA-MB-231 cells. Cells on coverslips were untreated (‘control’) or treated with VCSM13 helper phage or recombinant phages (VCSM13, 3D8 VL-M13 and hAY4 scFv-M13 at 10^12^ CFU for 6 h or TAT-M13 at 10^13^ CFU for 2 h) and then analyzed by confocal immunofluorescence microscopy using anti-pVIII antibody. Images show the merging of phages (*green*) and DAPI-stained nuclei (*blue*) at the centered single confocal section. **(B)** Confocal microscopic images of 3D8 VL-M13 and TAT-M13 phages internalized and localized within living cells. Cells treated with 10^12^ CFU of 3D8 VL-M13 for 6 h or 10^13^ CFU of TAT-M13 for 2 h were examined by confocal microscopy, as described in Materials and Methods. The images were obtained in 1-µm cuts of the Z-plane, starting from the baso-lateral surface (*bottom* image), moving toward the apex of the cells (*top* image). Blue color depicts DAPI-stained nuclei. Magnification, ×400; scale bar, 5 µm.
(TIF)

Figure S3 Effect of internalized phages on the cell viability (**A**) and morphological features (**B**) of HeLa cells. HeLa cells were treated at 37°C with medium (control), 10^12^ CFU of 3D8 VL-M13 for 6 h, or 10^13^ CFU of TAT-M13 for 2 h, washed out cell surface bound phages and incubated for 24 h prior to the MTT [3-(4,5-dimethylthiazol-2-yl)-2,5-diphenyltetrazolium bromide] assay and taking the images by phase contrast microscopy. Magnification, ×100; scale bar, 20 µm.
(TIF)

Figure S4 Cellular internalization of 3D8 VL-M13 and TAT-M13 phages in wild-type CHO-K1 cells and pgsD-677 and pgsA-745 mutants. Wild-type CHO-K1, the HS-deficient pgsD-677 (no HS, 3 times more CS), and HS/CS-deficient pgsA-745 (no proteoglycans) cells were treated with 10^12^ CFU of 3D8 VL-M13 for 6 h or 10^13^ CFU of TAT-M13 for 2 h and internalized phages were visualized by confocal microscopy. The images were obtained in 1-µm cuts of the Z-plane, starting from the baso-lateral surface (*bottom* image), moving toward the apex of the cells (*top* image). Blue color depicts DAPI-stained nuclei. Magnification, ×400; scale bar, 5 µm.
(TIF)

Figure S5 The separate and merged images to determine co-localization of internalized phages with endocytotic vesicle markers and subcellular organelle markers monitored at the indicated incubation time, the merged images of which are shown in Fig. 5B. Magnification, ×400; scale bar, 5 µm.
(TIF)
