# Supplementary figures and images for: Cellular Internalization Mechanism and Intracellular Trafficking of Filamentous M13 Phages Displaying a Cell-Penetrating Transbody and TAT Peptide
Source: PLoS One. 2012 Dec 14;7(12):e51813. doi: 10.1371/journal.pone.0051813 (PMC3522607; doi:10.1371/journal.pone.0051813)

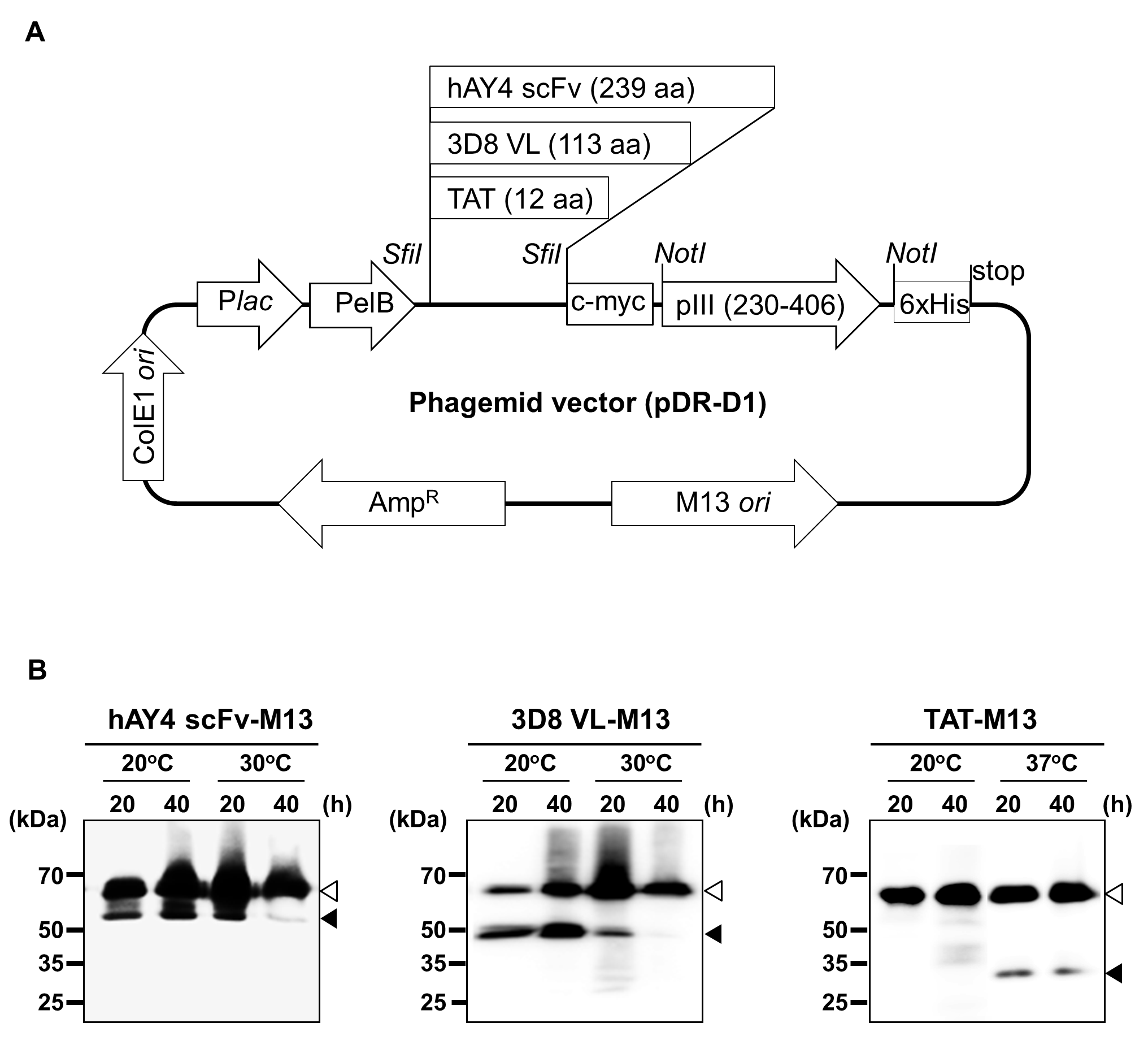

Supplement: Figure S1 — (A) Schematic diagram of pDR-D1-based phagemid vectors constructed for phage-surface display of 3D8 VL transbody, TAT peptide, and hAY4 scFv by fusion to the N-terminus of truncated minor coat protein pIII (residues 230–406). See text for details. (B) Western blotting of fusion protein display efficiency (insert-pIII) (filled arrow) versus full-length pIII (open arrow) from the VCSM13 helper phage on M13 filamentous phage particles, obtained from bacterial cultures at the indicated expression temperature and time. Phage particles were prepared by infecting phagemid-transformed ER2738 cells with the helper phage, adding 0.5 mM IPTG (TAT-M13) or 0 mM IPTG (3D8 VL-M13, hAY4 scFv-M13) and then incubating at 20°C, 30°C, or 37°C for 20 or 40 h. An equal titer (1010 CFU) of phage particles was used for Western blotting with anti-M13 pIII antibody for the detection of both pIII-fusion proteins (3D8 VL-pIII, and TAT-pIII, and hAY4 scFv-pIII) from the phagemid vectors and full-length pIII from the helper phage. The calculated molecular mass based on the amino acid sequence is ∼45.5 kDa for hAY4-pIII, ∼33.0 kDa for 3D8 VL-pIII, and 22.1 kDa for TAT-pIII fusion proteins and ∼65 kDa for full-length pIII protein from helper phage. (TIF) [file pone.0051813.s001.tif]

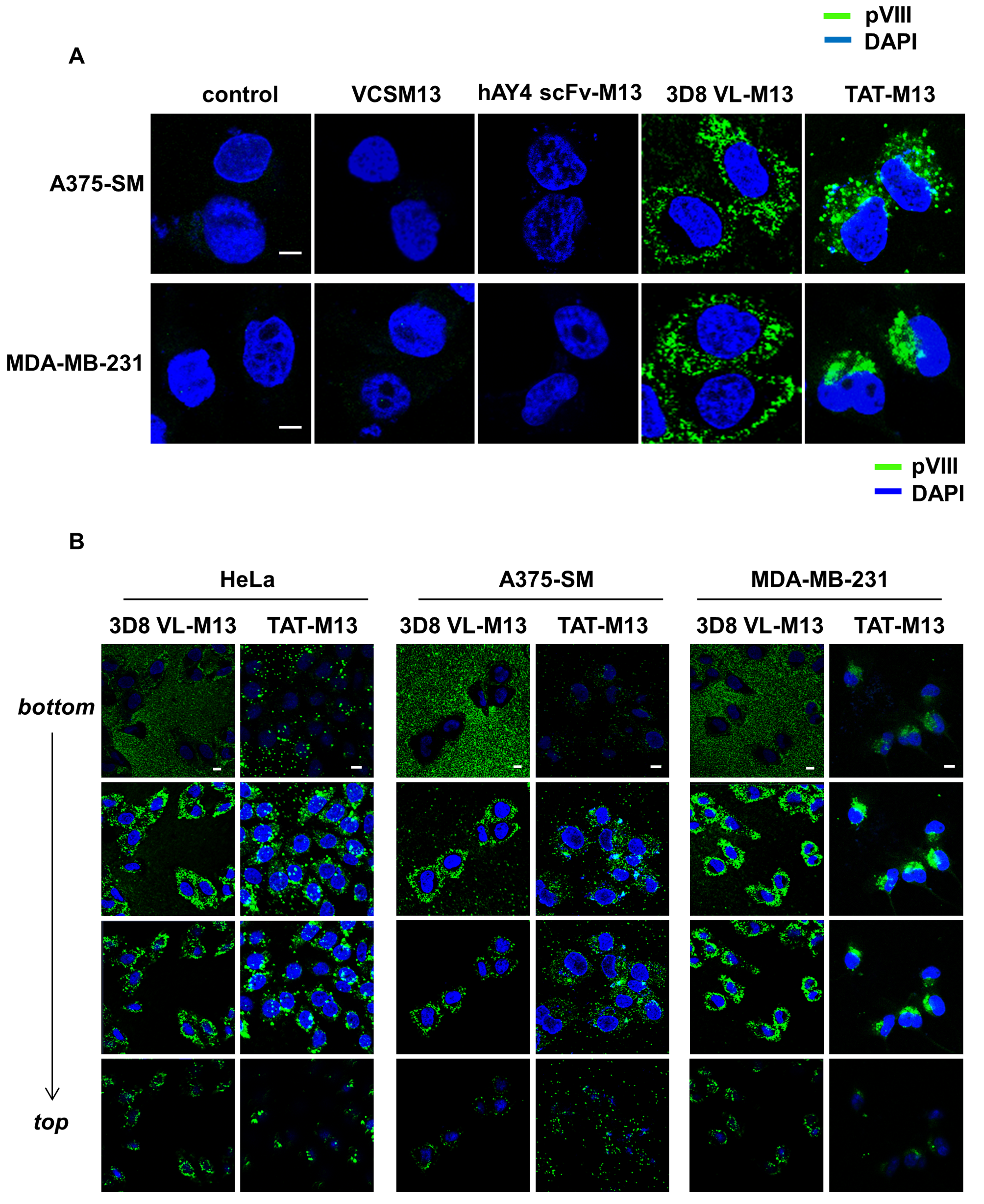

Supplement: Figure S2 — 3D8 VL-M13 and TAT-M13 phages internalize into living cells. (A) Internalization and subcellular localization of recombinant phages in A375-SM and MDA-MB-231 cells. Cells on coverslips were untreated (‘control’) or treated with VCSM13 helper phage or recombinant phages (VCSM13, 3D8 VL-M13 and hAY4 scFv-M13 at 1012 CFU for 6 h or TAT-M13 at 1013 CFU for 2 h) and then analyzed by confocal immunofluorescence microscopy using anti-pVIII antibody. Images show the merging of phages (green) and DAPI-stained nuclei (blue) at the centered single confocal section. (B) Confocal microscopic images of 3D8 VL-M13 and TAT-M13 phages internalized and localized within living cells. Cells treated with 1012 CFU of 3D8 VL-M13 for 6 h or 1013 CFU of TAT-M13 for 2 h were examined by confocal microscopy, as described in Materials and Methods. The images were obtained in 1-µm cuts of the Z-plane, starting from the baso-lateral surface (bottom image), moving toward the apex of the cells (top image). Blue color depicts DAPI-stained nuclei. Magnification, ×400; scale bar, 5 µm. (TIF) [file pone.0051813.s002.tif]

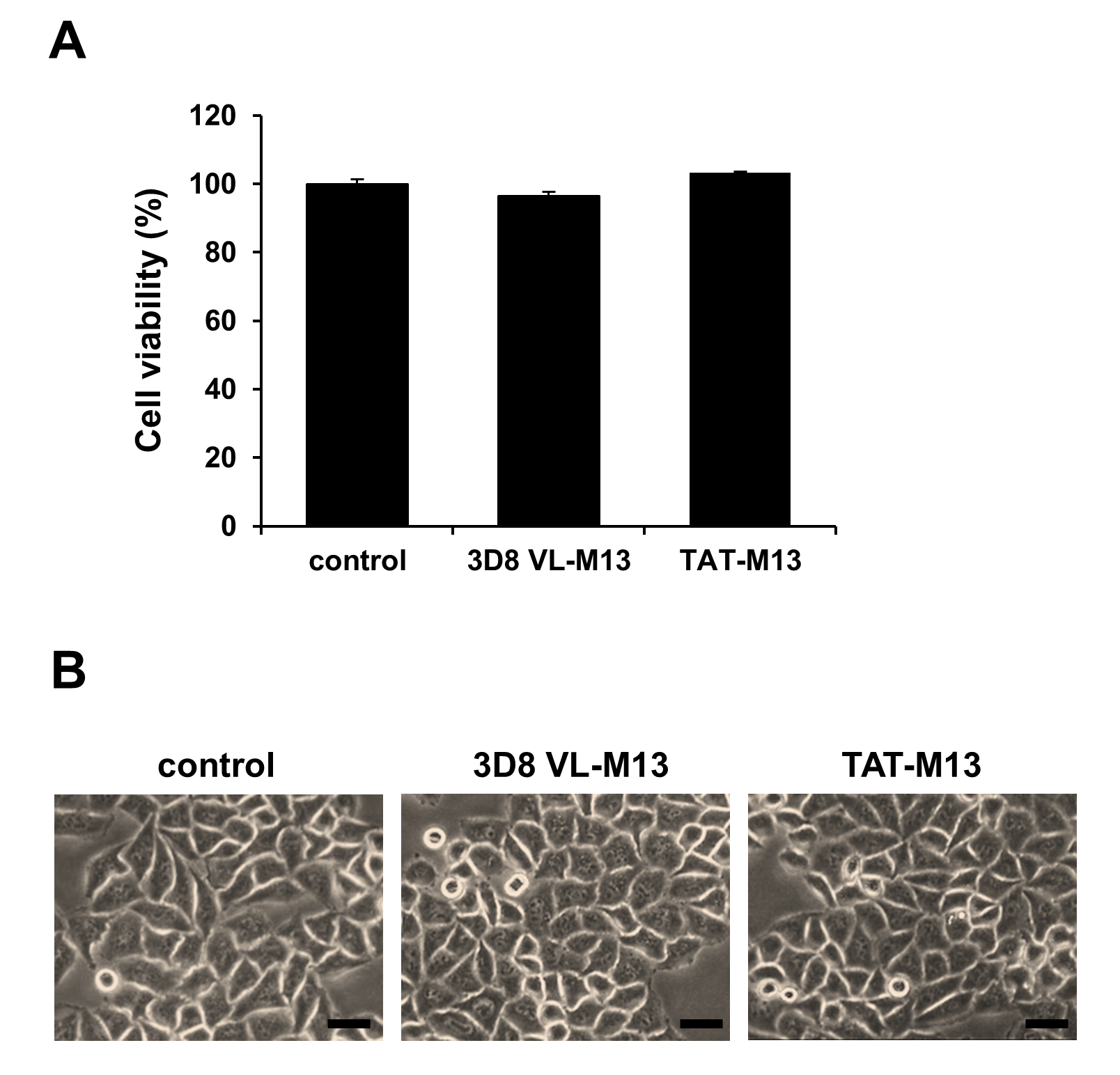

Supplement: Figure S3 — Effect of internalized phages on the cell viability (A) and morphological features (B) of HeLa cells. HeLa cells were treated at 37°C with medium (control), 1012 CFU of 3D8 VL-M13 for 6 h, or 1013 CFU of TAT-M13 for 2 h, washed out cell surface bound phages and incubated for 24 h prior to the MTT [3-(4,5-dimethylthiazol-2-yl)-2,5-diphenyltetrazolium bromide] assay and taking the images by phase contrast microscopy. Magnification, ×100; scale bar, 20 µm. (TIF) [file pone.0051813.s003.tif]

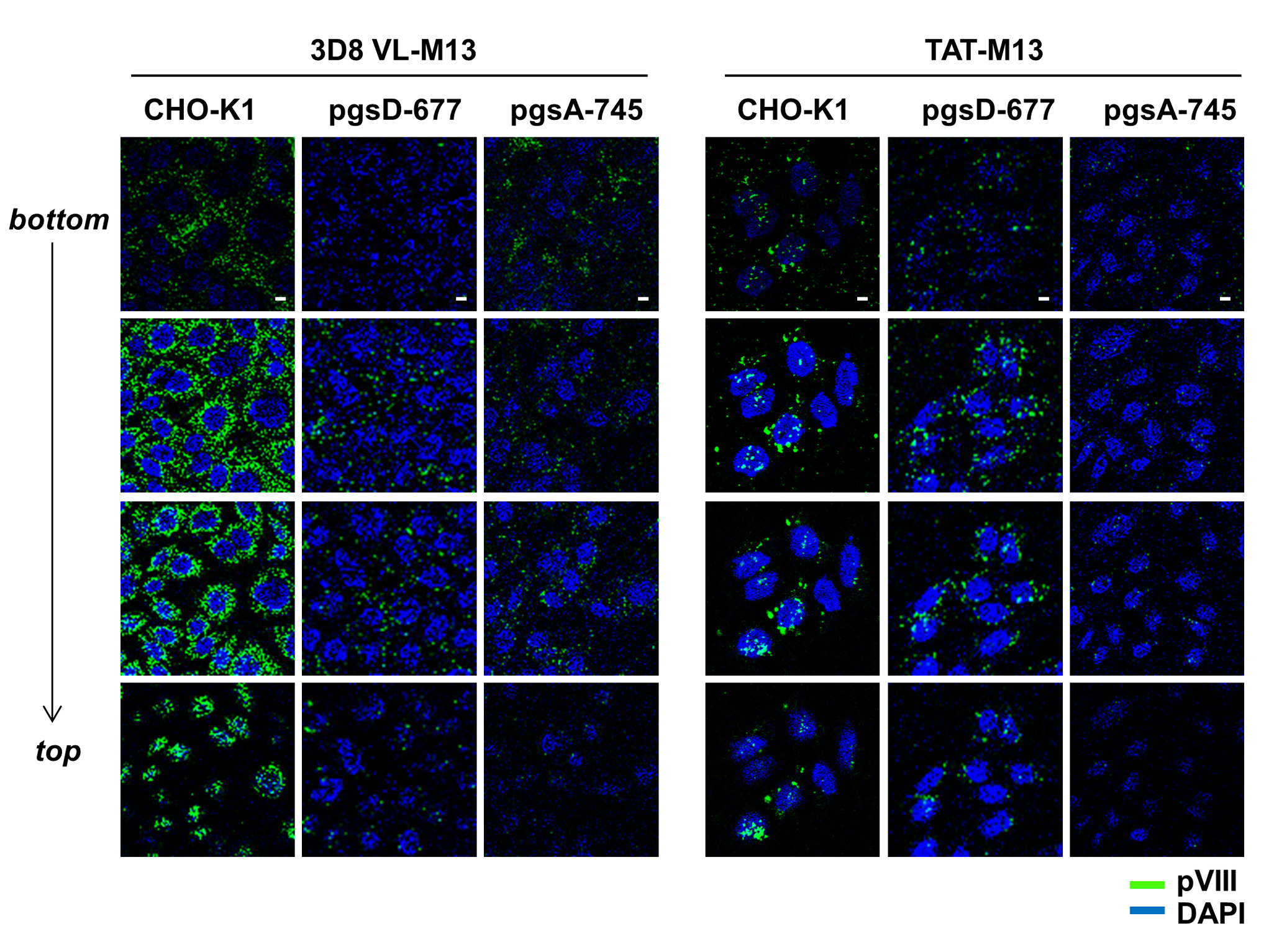

Supplement: Figure S4 — Cellular internalization of 3D8 VL-M13 and TAT-M13 phages in wild-type CHO-K1 cells and pgsD-677 and pgsA-745 mutants. Wild-type CHO-K1, the HS-deficient pgsD-677 (no HS, 3 times more CS), and HS/CS-deficient pgsA-745 (no proteoglycans) cells were treated with 1012 CFU of 3D8 VL-M13 for 6 h or 1013 CFU of TAT-M13 for 2 h and internalized phages were visualized by confocal microscopy. The images were obtained in 1-µm cuts of the Z-plane, starting from the baso-lateral surface (bottom image), moving toward the apex of the cells (top image). Blue color depicts DAPI-stained nuclei. Magnification, ×400; scale bar, 5 µm. (TIF) [file pone.0051813.s004.tif]

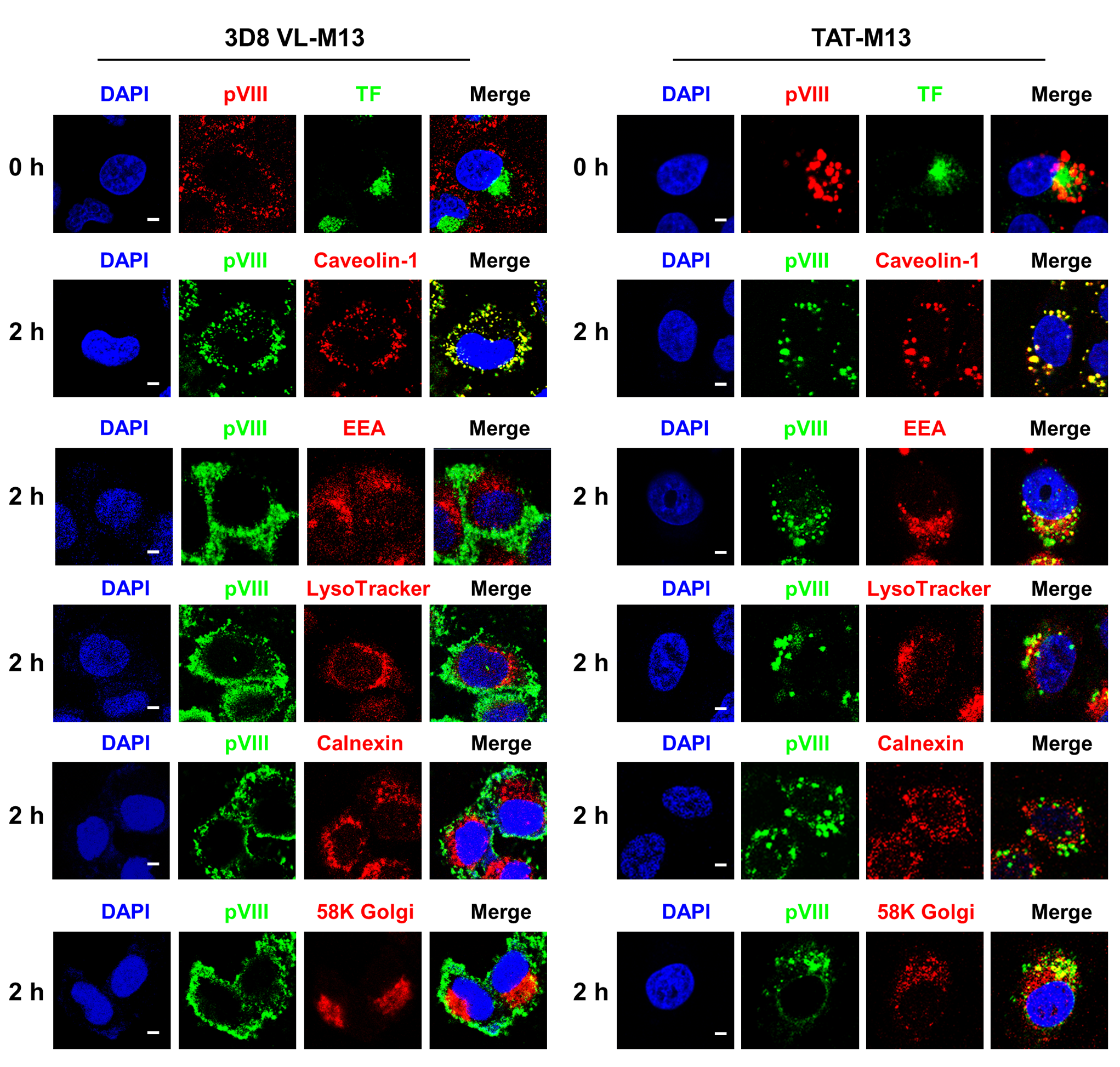

Supplement: Figure S5 — The separate and merged images to determine co-localization of internalized phages with endocytotic vesicle markers and subcellular organelle markers monitored at the indicated incubation time, the merged images of which are shown in Fig. 5B. Magnification, ×400; scale bar, 5 µm. (TIF) [file pone.0051813.s005.tif]
